# Utilization of *Metarhizium* as an insect biocontrol agent and a plant bioinoculant with special reference to Brazil

**DOI:** 10.3389/ffunb.2023.1276287

**Published:** 2023-12-21

**Authors:** Emily Mesquita, Shasha Hu, Tais B. Lima, Patricia Silva Golo, Michael J. Bidochka

**Affiliations:** ^1^ Department of Biological Sciences, Brock University, St. Catharines, ON, Canada; ^2^ Department of Animal Parasitology, Veterinary Institute, Federal Rural University of Rio de Janeiro, Seropedica, RJ, Brazil

**Keywords:** entomopathogenic fungi, endophytes, native isolates, rhizosphere-competence, integrated pest management (IPM)

## Abstract

Brazil has a long history of using biological control and has the largest program in sugarcane agriculture to which a biocontrol program has been applied. This achievement is at least partly due to the utilization of the entomopathogenic fungus *Metarhizium*. This well-known fungal genus exhibits pathogenicity against a broad range of arthropod hosts and has been used globally as a biocontrol agent. This fungus is also a root symbiont, and in this capacity, it is a plant growth promoter. However, this feature (i.e., as a plant symbiont) has yet to be fully explored and implemented in Brazil, although the number of reports demonstrating *Metarhizium*’s utility as a plant bioinoculant is increasing. The Brazilian bioproduct industry targets agricultural pests, and is limited to two *Metarhizium* species represented by four fungal isolates as active ingredients. Entomopathogenic fungi have also been successful in controlling arthropods of public health concern, as shown in their control of mosquitoes, which are vectors of diseases. The isolation of new indigenous *Metarhizium* isolates from a variety of substrates such as soil, insects, and plants shows the wide genetic diversity within this fungal genus. In this review, we emphasize the significance of *Metarhizium* spp. for the biological control of insects in Brazil. We also suggest that the experience and success of biological control with fungi in Brazil is an important resource for developing integrated pest management and sustainable strategies for pest control worldwide. Moreover, the future implementation prospects of species of *Metarhizium* being used as bioinoculants and possible new advances in the utility of this fungus are discussed.

## 1 Introduction

*Metarhizium* is a genus of entomopathogenic fungi in the family Clavicipitaceae, order Hypocreales. These fungi play multiple roles, as endophytes, saprobes, and pathogens of insects (Stone and Bidochka, 2020). Phylogenetic analysis showed that *Metarhizium* and *Pochonia chlamydosporia* form a monophyletic clade that evolved from the plant root symbionts *Claviceps* and *Epichloë* approximately 300 million years ago (MYA), and then diverged with pathogenic ability against nematodes and insects approximately 180 MYA (Sheng et al., 2022). In addition to this, there have been more recent studies carried out on entomopathogenic fungi as endophytes. Vega (2018) highlighted entomopathogenic fungal–plants interactions to integrate aspects of endophytism with insect pathogenesis in an applied sense. However, there is limited research on the effects of fungus-inoculated plants on arthropod pests in Brazil.

Based on the insect host range, *Metarhizium* species have been classified as generalists with broad host ranges and specialists with narrow host ranges (Gao et al., 2011; St Leger and Wang, 2020). For example, *Metarhizium acridum* was classified as a specialist pathogen restricted to Orthoptera (Wang et al., 2016), and generalists such as *Metarhizium anisopliae* infect a wide spectrum of insect hosts in the orders Lepidoptera, Coleoptera, Hemiptera, and Orthoptera (Balachander et al., 2009).

Both the generalist and specialist *Metarhizium* insect pathogens retain their ancestral ability to colonize plant roots (Moonjely and Bidochka, 2019). As plant symbionts, *Metarhizium* can improve plant growth (Ahmad et al., 2020; Hu et al., 2023), resist plant pathogens (Sasan and Bidochka, 2013; Gupta et al., 2022), and ameliorate salt stress (Chaudhary et al., 2023). As a bioremediator, *Metarhizium* can alleviate heavy metal pollution of mercury in soil and water (Wu et al., 2022) and enhance the cadmium efflux capacity of plants (Jiang et al., 2022).

With recent developments in the application of *Metarhizium* as a biocontrol agent, this review will focus on the utility and potential prospects of *Metarhizium* as a mycoinsecticide and plant bioinoculant in Brazil.

## 2 Genetic variation in Brazilian strains of *Metarhizium*

There is accumulating knowledge of the diversity and abundance of indigenous Brazilian strains (Mesquita et al., 2020; Couceiro et al., 2022; Diniz et al., 2021). According to Luz et al. (2019), *M. robertsii*, *Metarhizium humberi*, and *M. anisopliae* sensu stricto (s. str.) are abundant in Brazilian soils. The *Metarhizium* spp. diversity was explored using the nuclear intergenic region *MzIGS3* (Kepler and Rehner, 2013) collected from several Brazilian ecological biomes (Amazon, Caatinga, Cerrado, Atlantic Forest, and Pampa) in the dry and humid seasons (Riguetti Zanardo Botelho et al., 2019). This study showed that *Metarhizium* spp. occurrence is correlated with Brazilian biomes, that is, *M. robertsii* was the only species identified in the Pampas biome, while the taxonomically uncharacterized “*Metarhizium* sp. indet. 3” was identified mostly in the Caatinga biome. Currently, *M. humberi* (referred to as *Metarhizium* sp. indet. 1 in the study) is the most diverse haplotype, and, interestingly, the haplotypes identified from the Cerrado biome soils were entirely different from those identified from soils in the Amazon biome. The haplotype diversity of *M. humberi* has also been noted in previous studies (Rocha et al., 2013; Lopes et al., 2014; Rezende et al., 2015). According to Riguetti Zanardo Botelho et al. (2019), the Amazon biome was the only one where all *Metarhizium* spp. were identified, which is not unexpected as it holds great ecological diversity. These authors confirmed a great abundance of *M. robertsii* in soils, which is in agreement with Iwanicki et al. (2019). However, for *M. anisopliae*, it was suggested that in Brazil, the occurrence of this species was strongly correlated with arthropod hosts (Riguetti Zanardo Botelho et al., 2019; Rezende et al., 2015). The highest occurrence of *M. anisopliae* was detected by Rezende et al. (2015) in a diverse group of environments, that is, in soils from different biomes and insects.

The diversity of *Metarhizium* spp. identified in agricultural and non-agricultural habitats has revealed the predominance of *M. anisopliae* sensu lato (Mani 2 subclade) in sugarcane fields, while *M. humberi* (*Metarhizium* sp. indet. 1) was predominantly found in the undisturbed soils of native plant communities (Rezende et al., 2015). Moreover, regarding the natural occurrence of *Metarhizium* spp. in Brazilian soils, *M. brunneum* and *M. pingshaense* were detected in a strawberry field previously treated with two different *Metarhizium* spp. (Castro et al., 2016). Within these four species, the authors identified two additional *M. anisopliae* haplotypes, five *M. robertsii* haplotypes, and one each of *Metarhizium brunneum* and *Metarhizium pingshaense.*

## 3 Host range

The genetic and biochemical basis of the ability of *Metarhizium* to penetrate the insect cuticle is well known (Wang et al., 2016; Beys-da-Silva et al., 2020; Hong et al., 2023). After the conidium attaches to the insect cuticle, a germ tube is formed and terminates in an appressoria. From this structure, a penetration peg is formed, and through mechanical and enzymatic action (i.e., secreted proteases, chitinases, and lipases) (Zimmermann, 2007), the cuticle is breached, and the fungus reaches the arthropod hemolymph. Once inside the nutrient-rich hemocoel, the fungus grows and forms hyphal bodies termed blastospores. Blastospores can evade insect immune responses by producing a collagenous coat (Wang and St. Leger, 2006)) and producing an array of toxins and secondary metabolites that leads to arthropod death (Zimmermann, 2007).

Mycoinsecticides based on *M. anisopliae* s. str. in Brazil target the following insects: the spittlebugs *Mahanarva fimbriolata*, *Deois flavopicta*, and *Zulia entreriana* (Mascarin et al., 2019), while two products based on *Metarhizium rileyi* target the fall armyworm *Spodotera frugiperda* (Agrofit, 2023). However, *Metarhizium* spp. reportedly infect a broader range of insects in Brazil. Examples of the studies reporting the diversity of *Metarhizium* spp. in terms of their infecting a variety of insects are found in **Table 1**. For instance, the generalist *M. anisopliae* has been used to control arthropods important for public health such as *Aedes aegypti* larvae (Oliveira Barbosa Bitencourt et al., 2021; Gomes et al., 2023) and the Chagas disease vector *Triatoma infestans* (Rangel et al., 2020). More recently, less common *Metarhizium* spp. have been shown to infect other arthropod hosts. For example, *Metarhizium marquandii* demonstrated virulence against the termite *Nasutitermes* sp. (Diniz et al., 2021), and *Metarhizium braziliense* infected the corn leafhopper *Dalbulus maidis* (Hemiptera: Cicadellidae) naturally in maize crops (Souza et al., 2021). Furthermore, *Metarhizium* spp. infections in ticks have been reported, both in the field and in semi-field conditions, demonstrating biocontrol results for *Rhipicephalus microplus* (Camargo et al., 2016; Marciano et al., 2021; Carneiro et al., 2022) and *Rhipicephalus sanguineus* (Reis et al., 2008) in Brazil.

**Table 1:** Diversity of *Metarhizium* spp. and strains infecting different species of arthropods in Brazilian territory.

Metarhizium sp.	Isolate/strain	Host	Method of isolation	Reference
*Metarhizium anisopliae*	E9	*Glycaspis brimblecombei*	A	Domingues et al., 2022
	4.443 UFAC	*Nasutitermes* sp.	A	Diniz et al., 2021
	MaLCB255	*Ceratitis capitata*	A	Gava et al., 2021
	IBCB-196; IBCB-333; IBCB-348; IBCB-364; IBCB-383; IBCB-391; IBCB-425; ESALQ-E9	*Gonipterus platensis*	A	Jordan et al., 2021
	TOYOBO; Usina Paulista	*Thaumastocoris peregrinus*	A	Soliman et al., 2019
	IBCB 348	*Duponchelia fovealis*	A	Poitevin et al., 2018
*Metarhizium acridum*	CG 423	*Rhammatocerus schistocercoides*	A	Magalhaes et al., 2000
		*Tropidacris collaris, Cornops frenatum frenatum*, and *Parascopas obesus*	A	Schmidt et al., 2018
*Metarhizium robertsii*	RD-20.114	*Leucoptera coffeella*	A	Franzin et al., 2022
	ESALQ1426	*Dalbulus maidis*	A	Iwanicki et al., 2020
*Metarhizium brunneum*	RD-20.120	*Leucoptera coffeella*	A	Franzin et al., 2022
	ARSEF 4556; V275	*Aedes aegypti*	A	Prado et al., 2020
*Metarhizium rileyi*	CG381	*Spodoptera frugiperda*	A	Barros et al., 2021
	CG1153	*Anticarsia gemmatalis* and *Chrysodeixis includens*	A	Lopes et al., 2020
	UFMS 02; UFMS 03; UFMS 06; UFMS 07	*Helicoverpa armigera*	A	Loureiro et al., 2020
*Metarhizium humberi*	ESALQ 1374	Hemiptera: Cydnidae	B	Rezende et al., 2015
	CG814	*Hedypathes betulinus*	B	Lopes et al., 2014; Luz et al., 2019
	CG835	*Scaptocoris castanea*	B	Lopes et al., 2014; Luz et al., 2019
*Metarhizium lepidiotae*	CG1237	*Aegopsis balboceridus*	Unknown	Lopes et al., 2014
*Metarhizium pingshaense*	CG1091	*Cosmopolites sordidus*	Unknown	Lopes et al., 2014

A. In vitro.

B. Natural occurrence.

## 4 Habitat association

**Figure 1:**
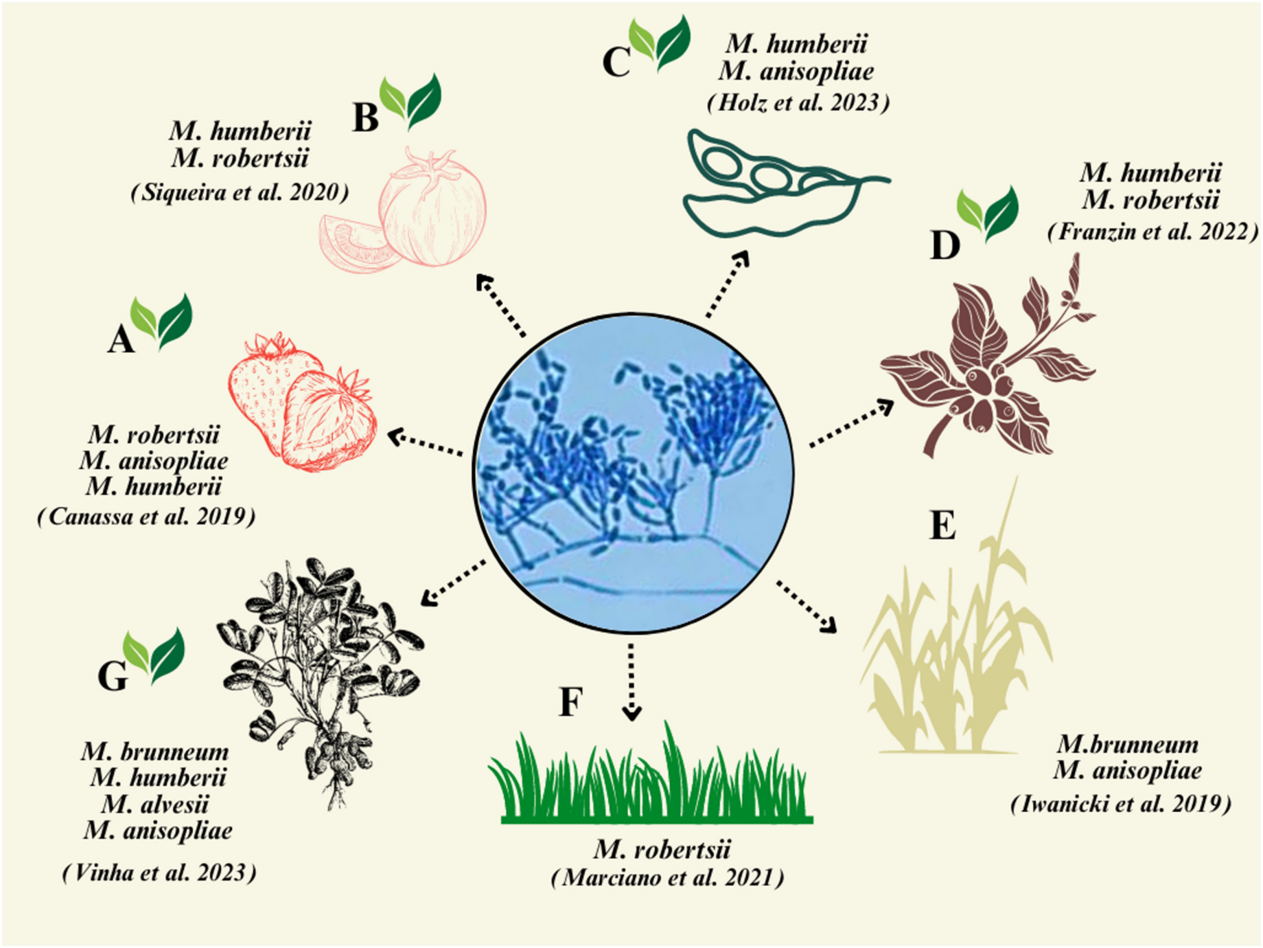
Representation of plant species studied for the isolation of *Metarhizium* spp. demonstrating the corresponding fungal species isolated from agricultural plants in Brazil. **(A)** Strawberry; **(B)** tomato; **(C)** soybean; **(D)** coffee; **(E)** sugarcane; **(F)** switchgrass; and **(G)** peanut. The green leaves indicate dicotyledons plants.

*Metarhizium* spp. are ecologically soil-borne fungi (Jaronski, 2007), and many have been demonstrated to be rhizosphere competent (Hu and St. Leger, 2002; Hu and Bidochka, 2021a), and these features can be exploited in biocontrol efforts (Bamisile et al., 2023). For example, in a sugarcane fields, an indigenous *M. anisopliae* strain—ESALQ 1604—persisted for up to 60 days after a soil drench application (Iwanicki et al., 2019). In a semi-field experiment, a native strain of *M. anisopliae* LCM S04 was shown to persist for up to 5 months post inoculation in soil in switchgrass pots (Mesquita et al., 2020). Additionally, in soil in which strawberry crops were grown, *Metarhizium* persistence was detected up to 1 year post treatment (Castro et al., 2016). According to Iwanicki et al. (2019), *M. brunneum* shows greater association with the rhizosphere than with bulk soil. In the same study, in addition to the spittlebugs that were infected with *M. brunneum* ESALQ 1604, endemic strains of *M. anisopliae* were found to infect up to 50% of the spittlebugs collected in the field. In Brazil, there is still limited information on the association of *Metarhizium* spp. with plant roots. It has been recovered from roots of strawberry (Canassa et al., 2020), sugarcane (Iwanicki et al., 2019), tomato (Siqueira et al., 2020), coffee (Franzin et al., 2022), grass (Marciano et al., 2021), peanut (Vinha et al., 2023), and soybean (Holz et al., 2023) (**Figure 1**). The recognition, connection, and relevance of these studies are shown in **Supplementary Figure S1**. Although not common, *Metarhizium* spp. were isolated in Goiás state from aquatic habitats (i.e., small- to medium-sized water bodies and lakes and rivers), where *A. aegypti* larvae were found (Rocha et al., 2022). The aquatic environment is suggested to be important for conidial recycling, as mosquito egg rafts are found on the surface of water bodies and mycosed mosquito larvae float on the surface of water bodies.

## 5 Development as a biological insect control agent in Brazil

One of the first reports of entomopathogenic fungi (probably of *Metarhizium*) killing crop insect pests in Brazil was done by Pestana (1923), who described sugarcane spittlebugs and their muscardine disease in Minas Gerais State (southeast Brazil). Because of the increasing occurrence of the sugarcane leaf spittlebug *Mahanarva posticata* in the northeastern states of Brazil in the 1960s and 1970s (Marques and Vilas Boas, 1978), along with reports of natural epizootics of the green muscardine disease in insects caused by *Metarhizium* across the country (Alves, 1998a), *Metarhizium* became a key subject in research and extension projects of several Brazilian government institutions (Li et al., 2010). An individual who was particularly instrumental in developing fungal biocontrol in Brazil was Dr. Donald W. Roberts (*in memoriam*), who received several Brazilian awards for his efforts and whose work is considered crucial to the success of the biological control narrative in Brazil. He was engaged in several projects in the country, especially at Embrapa Arroz e Feijão in Goiás state, where the work began, and supervised Brazilian students and researchers for many years. In recognition of his contributions to biocontrol efforts and to fundamental research, *M*. *robertsii* was named after him.

According to the Food and Agriculture Organization of the United Nations (FAO), Brazil is one of the world’s largest producers of agricultural and livestock commodities, including rice, barley, corn, soy, wheat, and beef (FAOSTAT, 2021). Brazil’s boom in agriculture production is claimed to have started with the “Green Revolution” (Nehring, 2022). Although using fertilizers and pesticides represents a major part of the crop production landscape, insecticide/acaricide resistance and pesticide residue are consequential environmental and health risks in Brazil and worldwide (Deguine et al., 2021.; Valentim et al., 2023). These concerns can be traced back many decades when the Brazilian government started seeking sustainable and safer alternatives for arthropod pest control, including the use of entomopathogenic fungi.

Since the mid 1900s, *Metarhizium* has been mass produced in Brazil, first using 1-L glass bottles that were later replaced by autoclavable plastic bags (Aquino et al., 1977; Alves, 1988b). Public and private research institutions have been working on developing more efficient and low-cost methods capable of large-scale, economical production of these fungi (Mascarin et al., 2015; Mascarin et al., 2019). In addition to low production costs, several factors are involved in the high acceptance of the use of entomopathogenic fungi for insect pest control in Brazil, including (i) effectiveness (Iwanicki et al., 2019), (ii) standard registration protocol, and (iii) on-farm production (on-farm production is defined as the production of beneficial microorganisms by growers exclusively for their own use) (Faria et al., 2023). Both solid-state (i.e., production of aerial conidia) and submerged liquid fermentations (i.e., production of hyphal bodies and/or blastospores) have been reported by Brazilian farmers. However, solid-state production is the most widely practiced form of fungal production (Faria et al., 2023). Only fungal-based products registered with the ANVISA that are manufactured or imported by companies authorized and licensed by the government may be commercialized in Brazil. Despite this, some of the current issues with mycoinsecticides in the country rely on the illegal production and distribution of non-registered products (Mascarin et al., 2019). These products usually do not undergo quality control during production, or shelf-life tests before distribution, resulting in low credibility. The reports of this condition have been addressed by Li et al. (2010). These authors also highlighted the program of pasture spittlebug control by *M. anisopliae* where the control rate was not satisfactory. Although most *Metarhizium*-based products are registered to control agriculture insect pests, some of these mycoinsecticides have been successfully tested against ticks under laboratory and field conditions (Camargo et al., 2014; Camargo et al., 2016; Nogueira et al., 2020). In addition to the existing commercial *Metarhizium* products, a wide variety of other *Metarhizium* fungal isolates have been tested against ticks in Brazil (Quinelato et al., 2012; Alves et al., 2017; Bernardo et al., 2018; Jones et al., 2021).

The use of native isolates of *Metarhizium* for research and technological development in Brazil is now regulated by the new biodiversity law established in 2015 (Law 13, 123). This law considers any microorganism isolated in the country as part of Brazilian genetic heritage, including *Metarhizium* spp. isolates (da Silva and de Oliveira, 2018). According to the law, researchers need to register their access to *Metarhizium* species in an online system (National System for the Management of Genetic Heritage and Associated Traditional Knowledge—SisGen) before disseminating results, shipment, and application for intellectual propriety. Some authors claim that there are positive aspects of this law (these pertain to its protection of Brazilian biodiversity), whereas others have expressed concern about research bureaucratization and barriers to basic research and international collaboration (da Silva and de Oliveira, 2018; Alves et al., 2018).

## 6 Development as a plant bioinoculant

*Metarhizium* spp. are reported as plant growth promoters, root colonizers, and endophytes (Garcia et al., 2011; Wyrebek et al., 2011; Hu and Bidochka, 2021a), and have the ability to protect plants from phytopathogenic fungi and can affect insect pest feeding and oviposition behavior in inoculated plants (Sasan and Bidochka, 2013; Canassa et al., 2020; Hao et al., 2021). Plant recognition of *Metarhizium* spp. as a beneficial symbiont may occur through the downregulation of plant defense mechanisms (Hu and Bidochka, 2021b) and decreases in plant oxidative responses, for example, soybean under salinity stress (Khan et al., 2012). However, studies of plant association with *Metarhizium* are more recent than the long-term studies of these entomopathogenic fungi in insect pest control programs. In Brazil, recent publications have started to analyze the diversity of native Brazilian strains in association with soil and plants and assess the potential effects on plant health and growth. While the development of these fungi as plant bioinoculants is still in its early stages, such research efforts are essential to study the feasibility and future use of *Metarhizium* spp. for plant growth promotion.

Seed treatment and direct soil drenching are usually successful in establishing fungi as rhizoplane colonizers and as endophytes. In the coffee plant (*Coffea arabica*), a study by Franzin et al. (2022) found that a soil drench with conidial suspensions promoted plant growth and provided protection against the coffee leaf miner (*Leucoptera coffeella*) using the Brazilian isolates *M. robertsii* (RD-20.114) and *M. brunneum* (RD-20.120) (Franzin et al., 2022). The application of *M. robertsii* significantly increased the coffee leaf area and suppressed foliar damage by the coffee leaf miner. This study reported that female insects that emerged from the plants inoculated with *M. robertsii* produced half the number of eggs produced by those from control plants. The inoculation method was successful in establishing both species in the root area for up to 43 days, although this study did not differentiate between rhizoplane soil or plant tissue when assessing colonization. Soil inoculation with Brazilian isolates of *M. robertsii* and *M. humberi* also promoted growth in tomato (*Solanum lycopersicum* L., ‘Micro Tom’ variety), with significant effects reported for *M. robertsii* ESALQ 1635 (Siqueira et al., 2020). After 30 days, the plants inoculated with *M. robertsii* showed a significant increase in traits such as height, root length, root weight, and overall biomass compared with controls, as well as a larger number of flowers and increased fruit weight. Both species were retrieved from rhizoplane soils and, interestingly, were also found to colonize the plant endophytically in all tissues, although a higher level of colonization was observed in the roots. This has also been observed in some studies that reisolated *Metarhizium* spp. from aboveground tissues following plant inoculations, although usually at lower levels than in the root region (Garcia et al., 2011; Jaber and Enkerli, 2016; Ahmad et al., 2020). Siqueira et al. (2020) also analyzed these strains of *Metarhizium* for certain biochemical traits and observed that the levels of phosphorus solubilization and plant hormone indole-3-acetic acid (IAA) production were comparable to those observed in a commercial strain of *Trichoderma harzianum*. *T. harzianum* is a well-known plant growth promoter that is widely used in Brazil, mostly as a biological control agent for its antagonistic interactions with soil-dwelling phytopathogenic fungi and nematodes (Nascimento et al., 2022).

In addition to *Trichoderma* spp., *Metarhizium* spp. have also been reported for their antagonistic performance against other fungi. Holz et al. (2023) recently described the ability of two Brazilian *Metarhizium* isolates to protect host plants from the fungal pathogen*, Phakopsora pachyrhizi*, the causal agent of Asian soybean rust. Soil drench applications of the *M. robertsii* Brazilian strain MHBR-03 later resulted in a significant decrease in rust disease symptoms in soybean following the foliar spray application of *P. pachyrhizi* spores on plants. Interestingly, foliar applications of *Metarhizium* cell-free culture filtrates also showed a degree of protection against the symptoms of rust *in vivo* and affected *P. pachyrhizi* development *in vitro*, which is an indication that metabolites produced by *M. robertsii* and released into the aqueous media could be responsible for rust inhibition, either directly or indirectly by activating plant defense mechanisms. Although further investigation was not performed to elucidate this particular finding, entomopathogenic fungi are known for their production of secondary metabolites that can potentially inhibit phytopathogens (Lozano-Tovar et al., 2017; Wei et al., 2022).

In addition to soil drench, seed treatments have been reported as an effective method for the application of *Metarhizium* spp. as plant bioinoculants. Bean (*Phaseolus vulgaris*) seed inoculations with a Brazilian strain of *M. robertsii* (ESALQ 1622) resulted in increased plant growth, root area, and aerial weight in treated plants (Canassa et al., 2019). It was possible to reisolate *M. robertsii* only from root rhizoplane with low levels of endophytic colonization. *M. robertsii* is a well-known plant rhizoplane colonizer and endophyte (Liao et al., 2014; Behie et al., 2015; Barelli et al., 2018). This fungus also promoted indirect protection against the spider mite (*Tetranychus urticae*), a primary pest mite commonly found in beans and other crops, which had a lower rate of population growth in inoculated plants (Canassa et al., 2019). The same species of mite was also suppressed in strawberry plants (*Fragaria* × *ananassa*) following root inoculation with *Metarhizium* spp., with lower oviposition rates by female mites (Canassa et al., 2020). The authors described plant improvements associated with fungal inoculations, such as increased fruit yield and overall plant growth, with effects that varied among different species and isolates. Once more, a comparison with commercial strains showed that the plant growth-promoting abilities of several *Metarhizium* isolates were comparable to those of *T. harzianum*, *Bacillus subtilis*, and *Bacillus licheniformis*, which are active ingredients in commercial agricultural products.

A novel application strategy using seed treatments to establish *Metarhizium* as a plant bioinoculant was investigated by Lira et al. (2020), who selected three Brazilian isolates for their ability to produce microsclerotia, and used this propagule for corn plant colonization. Microsclerotia are resistant fungal structures which, given their hardiness and ability to withstand desiccation, have been studied as potential active ingredients in microbial bioinoculants in a variety of fungal species (Kobori et al., 2015; Huarte-Bonnet et al., 2019; Marciano et al., 2021; Rodrigues et al., 2021). Seed coating using microsclerotia granules with Brazilian isolates of *M. humberi*, *M. anisopliae*, and *M. robertsii* influenced plant traits, such as root length, plant dry weight (Lira et al., 2020), and mortality of the fall armyworm (*S. frugiperda*) larvae when feeding on treated plants. However, fungal inoculation did not affect the mortality of the leafhopper, *D. maidis*, and this could be due to the differences in the feeding behaviors of the two insects. Leafhoppers are Hemiptera, with sucking mouth parts, while armyworm larvae are Lepidoptera, with chewing mouth parts. This study highlighted the potential of microsclerotia not only in biopesticide formulations, but also in seed treatments aiming to establish fungal colonization in host plants.

As more studies investigate *Metarhizium* spp. with a focus on their relationship with host plants, the potential of these fungi beyond their use as entomopathogens is being revealed. Different species and strains of *Metarhizium* interact differently with plant hosts, which shows the importance of strain selection for specific objectives when developing novel biological control tools. The majority of *Metarhizium*-based products commercially available in Brazil are used specifically as topical sprays against insect pests, meaning that they may not be optimal candidates as plant bioinoculants; however, this assertion is currently underexplored.

With six distinct biomes and a vast land area, Brazil has a huge variety of naturally occurring strains of *Metarhizium* both in natural and agricultural areas (Rocha et al., 2013; Riguetti Zanardo Botelho et al., 2019; Couceiro et al., 2022), many of which have been isolated from soils and in association with plants, and which could therefore be explored for their potential as plant growth promoters. The results described earlier in this section show the high levels of genetic diversity within *M. robertsii*, *M. humberi*, and *M. anisopliae*, and exemplify how this genetic variability could be explored by Brazilian biopesticide producers for the development of *Metarhizium* as a plant bioinoculant.

## 7 Commercial products in Brazil

The registration of biological products in Brazil is regulated by the Brazilian Health and Surveillance Agency (ANVISA) under resolution RDC 55/2010 (Brazil Official Union Diary). According to the Brazilian Ministry of Agriculture, Livestock and Supply, there are 91 registered products based on *Metarhizium* spp. (alone or with other biological agents) (Agrofit, 2023). Most of these products have *M. anisopliae* as their active ingredient, that is, *M. anisopliae* IBCB 425 has 87 registered products, *M. rileyi* CCT7771 has two products, and *M. anisopliae* IBCB 348 and *M. anisopliae* have one product each. These products are essentially directed to the control of spittlebug species, as previously mentioned. The use of *Metarhizium* against sugarcane insect pests in Brazil is considered one of the most successful biological control programs in the world, with millions of hectares treated annually (Parra, 2014; Iwanicki et al., 2019; Mascarin et al., 2019). According to the Brazilian Ministry of Agriculture, Livestock and Supply there are 3,293 registered pesticides. Of these, 593 are based on biological organisms (i.e., microbiological insecticide/acaricide/bactericide/fungicide/nematicide or other biological control agents), which constitute approximately 18% of the pesticide market. Mycoinsecticides and mycoacaricides based on *Metarhizium* and *Beauveria* spp. represent approximately 6% of the pesticide market (198 products). Although this review focused on *Metarhizium* spp., *Beauveria bassiana* products constitute up to 101 registered pesticide products according to government data (Agrofit, 2023). Commercial products in Brazil must rely on formulation techniques and consider environmental conditions that may impair fungal biology, such as temperature, UV radiation, and humidity (Acheampong et al., 2020a; Acheampong et al., 2020b). Although the microbial control business in Brazil is continuously increasing, available products in the market are mainly based on wettable powder formulations and the addition of oil as an adjuvant (Faria and Wraight, 2007; Mascarin et al., 2019). Unfortunately, industry places little emphasis on shelf life and technologies that improve insect pathogenicity and delivery. There is a perceived lack of investment and interest in formulation research to boost efficacy, although the relevance of this improvement has already been reported (Vemmer and Patel, 2013; Iwanicki et al., 2021; Marciano et al., 2021; Meirelles et al., 2023). Nonetheless, this is not peculiar to Brazil, especially due to the time required to approve a novel formulation. Biocontrol companies worldwide follow similar patterns. In addition to this, more recently, a report on the product ATTRACAP^®^ (BIOCARE GmbH, Germany) described its efficacy against wireworms (Coleoptera: Elateridae) in an “attract-and-kill” strategy (Gvozdenac et al., 2022). This granular formulation is a *M. brunneum*-based bead constituted with alginate (polymer), starch (nutrient), and *Saccharomyces cerevisiae* (CO_2_ source) (Working Group Patel, 2023).

The majority of the mass production of *Metarhizium* is done using solid substrate fermentation with cereal grains and rice, with the aim of producing high yields of aerial conidia (Jaronski, 2013; Mascarin et al., 2019; Jaronski, 2022; Rangel et al., 2023). In addition to this, liquid culture fermentation yielding blastospores and hyphal bodies has been studied, as it has a better cost-to-benefit ratio and faster production (Mascarin et al., 2015; Mascarin et al., 2019). The biggest concern around the use of blastospores is their suggested low tolerance to abiotic factors. However, Bernardo et al. (2020) have compared conidia and blastospores of *Metarhizium* spp. and *Beauveria bassiana* with respect to susceptibility to UV-B and heat stress. Their study showed that blastospores of *B. bassiana* CG 307 exhibited higher tolerance to heat than conidia, while *M. robertsii* and *M. anisopliae* blastospores and conidia were equally tolerant to UV-B.

## 8 Conclusions

Brazil has a well-established agricultural market and is an international leader in insect biocontrol, particularly with regard to sugarcane. However, there is a paucity of information on the use of *Metarhizium* as a plant growth promoter. Given Brazil’s geography and biome diversity, there is an abundance of and diversity within Brazilian isolates of *Metarhizium* that is currently underexplored. The Brazilian government has astutely protected this diversity, which could also serve as a potential export resource and could benefit the agricultural market in neighboring countries in South America. In this study, we highlighted Brazilian products commercially available based on *Metarhizium* that rely mostly on only four *Metarhizium* isolates among the 91 registered products. This approach, however, underrepresents the variety of species and underexplored genetic diversity found in Brazil. Farmers and bioproduct business owners could better assess and potentially exploit the diversity of *Metarhizium* not only as insect pathogens but also as plant bioinoculants. Moreover, the widespread use of biological control agents and bioinoculants for both pest control and plant improvement could benefit Brazil’s agroindustry. According to Guida et al. (2018), Brazil has been the number one user of agrochemicals globally since 2008. The application of bioproducts could support and diversify agroindustry in Brazil as well as affording benefits to human health and a sustainable environment.

## Author contributions

EM: Writing – original draft, Writing – review & editing. SH: Writing – original draft. TL: Writing – original draft. PG: Writing – original draft, Writing – review & editing. MB: Conceptualization, Writing – original draft, Writing – review & editing.

## Supplementary material

Supplementary Figure 1 Map of citations per paper according to the studies presented in **Table 1** and **Figure 1**. Each circle correlates to one paper. The grouped circles represent the connection of papers by similarity. The number of citations and importance of papers are demonstrated by the size of the circle and the color demonstrated in the legend.
