# Supplementary figures and images for: Utilization of Metarhizium as an insect biocontrol agent and a plant bioinoculant with special reference to Brazil
Source: Front Fungal Biol. 2023 Dec 21;4:1276287. doi: 10.3389/ffunb.2023.1276287 (PMC10768067; doi:10.3389/ffunb.2023.1276287)

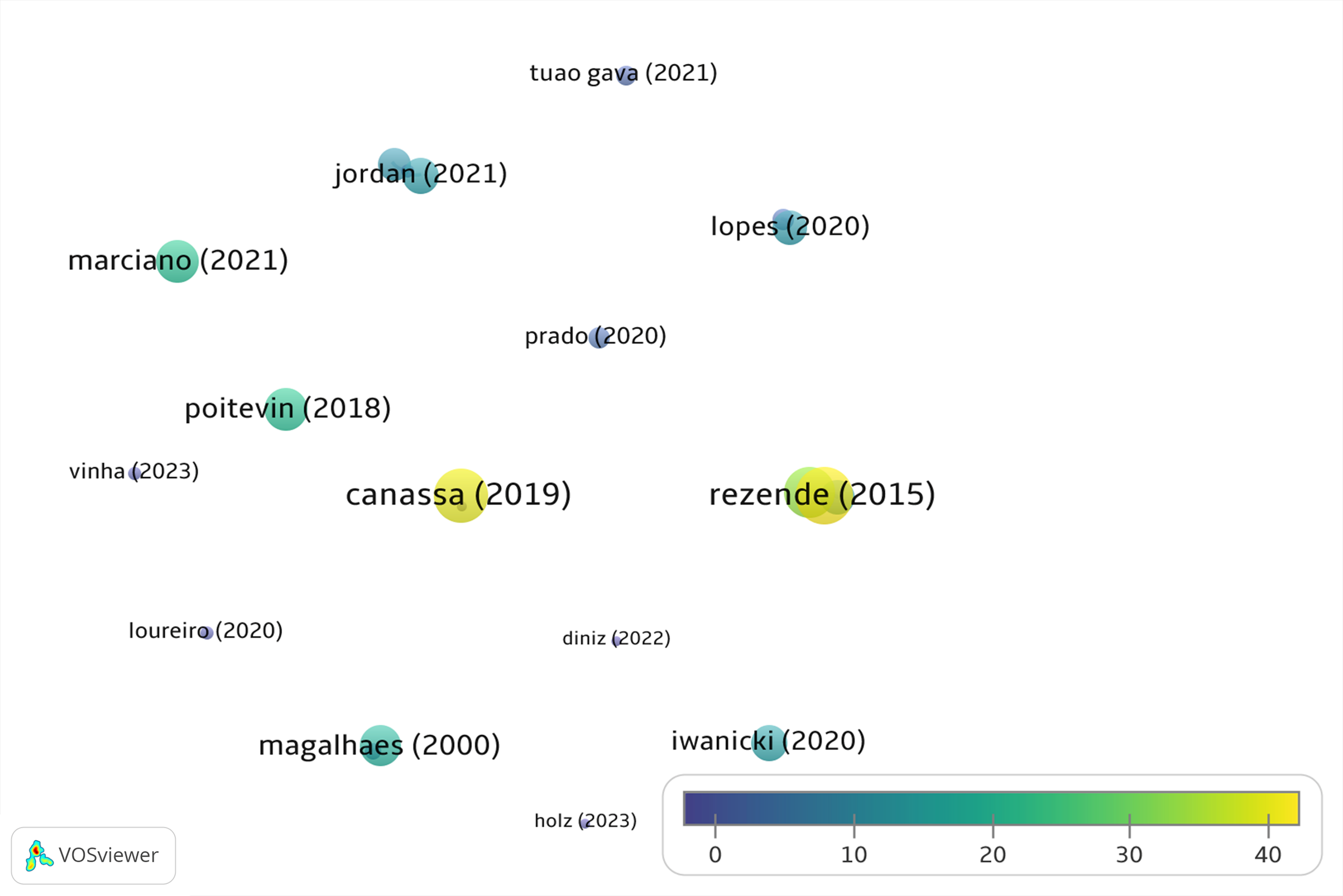

Supplement: Supplementary Figure 1 — Map of citations per paper according to the studies presented in Table 1 and Figure 1 . Each circle correlates to one paper. The grouped circles represent the connection of papers by similarity. The number of citations and importance of papers are demonstrated by the size of the circle and the color demonstrated in the legend. [file Image_1.tif]
